# A novel mechanism of rotavirus infection involving purinergic signaling

**DOI:** 10.1007/s11302-021-09773-y

**Published:** 2021-02-18

**Authors:** Wilmarie Morales-Soto, Brian D. Gulbransen

**Affiliations:** 1 Neuroscience Graduate Program, Biomedical and Physical Sciences Building, Michigan State University, 567 Wilson Rd, East Lansing, MI 48824 USA; 2 Department of Physiology, Biomedical and Physical Sciences Building, Michigan State University, 567 Wilson Rd, East Lansing, MI 48824 USA

## Article Summary

Rotavirus (RV) infection causes life-threatening diarrhea, despite only infecting a limited number of intestinal cells. A long-held concept of how this occurs is that RV infection induces the release of potent signaling molecules that can dysregulate neighboring, uninfected cells. However, the mechanisms of RV-induced signaling changes are multifactorial and incompletely understood. Ghang-Graham et al*.* (2020) addressed this issue in their study which was published in the journal *Science*. Here, by combining sophisticated long-term live fluorescent imaging techniques and CRISPR technology in simian- and human-derived cell cultures, the authors show that RV-infected cells signal to surrounding uninfected cells through a purinergic signaling pathway. The data show that RV infection induces intercellular calcium (Ca^2+^) waves (ICWs) that propagate between infected and neighboring uninfected intestinal epithelial cells. This process is driven by adenosine diphosphate (ADP) release from RV-infected cells and its subsequent interaction with P2Y_1_ receptors expressed by neighboring cells. This mechanism appears to be independent of traditional RV-induced signaling pathways that involve paracrine signaling molecules such as prostaglandin E-2 (PGE2), nitric oxide (NO), or RV enterotoxin nonstructural protein 4 (NSP4). Ca^2+^ responses in surrounding non-infected cells require P2Y_1_ receptors, and disrupting intercellular purinergic through P2Y_1_ receptors decreases the rate of RV replication and expression levels of interleukin-1α (IL-1α), cyclooxygenase-2 (COX-2), and inducible nitric oxide synthase (iNOS) mRNA following RV infection. Purinergic signaling through P2Y_1_ receptors also contributes to diarrhea by stimulating serotonin (5-HT) secretion from RV-infected cells, and small molecule inhibitors of P2Y_1_ receptors reduced diarrhea severity in infected animals. Thus, P2Y_1_-ADP signaling is a promising target for future RV infection therapies.

## Commentary

RV is among the most severe, life-threating diarrhea-inducing viruses, and it affects millions of children worldwide. RV causes these effects by infecting enterocytes and enteroendocrine cells in the small intestine in which RV replicates by disrupting host cell calcium signaling. However, not all susceptible cells become infected, and how they are involved in RV-disease progression has remained an open question. RV induces major alterations to host cell homeostasis which result in the release of potent paracrine signaling molecules including enterotoxin NSP4, PGE2, and NO (see Chang-Graham et al*.* [1] for references). These paracrine signals are linked to dysregulated serotonergic signaling and enteric modulated secretory function in the gastrointestinal (GI) tract [2, 3]. Thus, a long-standing hypothesis is that neighboring uninfected or bystander cells contribute to RV disease pathogenesis by responding to these viral and cellular factors that are secreted by infected cells.

Functional cell-to-cell signaling had not been directly observed during RV infection prior to the study by Chang-Graham et al., and the signaling pathways involved in this process were incompletely understood. Chang-Graham et al*.* addressed this issue by studying intercellular signaling between RV-infected and bystander African green monkey epithelial kidney cells expressing the genetically encoded calcium indicator GCaMP5G or GCaMP6s. The authors observed large increases in cytosolic calcium in host cells following RV infection which were followed by increases in cytosolic Ca^2+^ in uninfected neighboring cells. ICWs such as these are considered important for the spatial coordination of cellular functions [4]. Antibody staining for the RV antigen and reporter assays in cells infected with a recombinant RV strain expressing mRuby3 confirmed that these effects on bystander cells were not due to infection but instead propagated via intercellular signaling.

Virally encoded NSP4 is responsible for inducing ICWs in RV-infected cells and is, therefore, the usual suspect for disease onset and progression [1]. Chang-Graham et al. used short hairpin RNA to reduce NSP4 and found that NSP4 was indeed necessary for the initial increase in intracellular Ca^2+^ in infected cells. However, anti-NSP4 antibodies were not able to reduce ICWs, suggesting that enterotoxin NSP4 was not required for ICW propagation to neighboring cells. Instead, the authors found that the extracellular messenger and receptor complex responsible for mediating RV-induced ICWs were ADP acting on P2Y_1_ receptors. This conclusion was supported by data showing that apyrase, an enzyme that degrades extracellular ADP and adenosine triphosphate, decreased Ca^2+^ responses in uninfected bystander cells. Further, by reducing P2Y_1_ receptor signaling with the antagonist BPTU or by CRISPR-Cas9-mediated knockdown of *P2RY1* prevented the formation of ICWs but did not prevent infection. Similar results were observed when the authors tested a different RV variant strain, which suggests that purinergic signaling is a common mechanism induced by multiple strains of RV. These effects were independent of previously identified RV-induced signaling molecules, and the addition of PGE2 and NO did not elicit Ca^2+^ fluctuations or directly transmit ICWs. The authors replicated these findings in human jejunal-derived intestinal enteroids expressing GCaMP and determined that human enterocytes utilize ADP-P2Y_1_ Ca^2+^-induced signaling under basal conditions, and that this is later exploited during RV infection. These findings are in accordance with the pivotal role of purinergic signaling in regulating gut homeostasis and pathology [5].

In addition to inducing ICWs, purinergic signaling was also necessary for the replication rate of RV and induced the production of cellular factors canonically known to play a role in the development of RV-associated pathology. Inhibiting ADP-P2Y_1_ purinergic signaling significantly decreased the expression of IL-1α, COX-2, and iNOS mRNA, which function to produce PGE2 and NO and subsequently activate fluid secretion process leading to diarrhea [1]. Furthermore, disrupting purinergic signaling reduced fluid secretion in intestinal enteroids and decreased RV-induced 5-HT production. This was an exciting finding, given that 5-HT is a key neurotransmitter in intestinal physiology and is known to contribute to the severe water loss within the intestinal epithelium and diarrhea associated with RV infection [2]. Importantly, the authors found that blocking P2Y_1_ receptors with BPTU reduced diarrhea severity in neonatal mice and shortened recovery times.

This study provides a detailed mechanism that explains how RV induces pathology through intercellular purinergic signaling. Perhaps, the most important observation by Chang-Graham et al. is that blocking the formation of ICWs in bystander cells through the inhibition of P2Y_1_ receptors decreases RV-induced diarrhea severity in mice. Several drugs targeting P2Y receptors have emerged as candidate therapies for cardiovascular and inflammatory diseases and neurodegeneration [6]. The results of this study suggest that these or related compounds could be promising therapeutics that limit RV disease progression.

Another exciting development from this paper was the identification of the integral role that uninfected cells play in disease progression. Historically, infectious disease research has primarily focused on infected cells. However, Chang-Graham et al*.*’s work suggests that expanding research in this area to include noninfected neighboring cell populations would improve the mechanistic understanding of disease progression and ultimately, drug development. This is certainly true when it comes to defining the molecular mechanism of enteric pathogen-induced pathologies. For example, norovirus is known to require very few viral particles to induce severe pathology [7], and it is possible that the progression of the disease may very well involve dysregulated signaling from uninfected cells in response to signals from the host cell. In their recent analysis, Stanifer and Boulant [7] highlight how this mechanism should also be considered when investigating GI pathology in the novel coronavirus disease 2019 (COVID-19). Severe acute respiratory syndrome coronavirus 2 (SARS-CoV-2) replication also occurs within the human intestinal epithelium and, although the number of infected cells found in biopsies appears to be very few, uninfected neighboring cells may contribute to the progression of GI symptoms. Chang-Graham et al. provides a unifying view of how rotavirus induces pathologies and has identified mechanisms that expanded the gamut for drug discovery research and will propel the field forward.

## Data Availability

NA
